# Identification of NPB, NPW and Their Receptor in the Rat Heart

**DOI:** 10.3390/ijms21217827

**Published:** 2020-10-22

**Authors:** Shashank Pandey, Zdenek Tuma, Elisa Peroni, Olivier Monasson, Anna Maria Papini, Magdalena Chottova Dvorakova

**Affiliations:** 1 Department of Pharmacology and Toxicology, Faculty of Medicine in Pilsen, Charles University, 323 00 Pilsen, Czech Republic; shashank.pandey@lfp.cuni.cz; 2 Biomedical Center, Faculty of Medicine in Pilsen, Charles University, 323 00 Pilsen, Czech Republic; zdenek.tuma@lfp.cuni.cz; 3 PeptLab@UCP Platform and CNRS BioCIS, CY Cergy Paris Université, 950 31 Cergy-Pontoise CEDEX, France; elisa.peroni@u-cergy.fr (E.P.); olivier.monasson@u-cergy.fr (O.M.); annamaria.papini@unifi.it (A.M.P.); 4 Interdepartmental Research Unit of Peptide and Protein Chemistry and Biology, Department of Chemistry “Ugo Schiff”, University of Florence, 500 19 Sesto Fiorentino, Italy; 5 Department of Physiology, Faculty of Medicine in Pilsen, Charles University, 323 00 Pilsen, Czech Republic

**Keywords:** neuropeptide B, neuropeptide W, NPBW receptor 1, RT-qPCR, Western blot, heart

## Abstract

Members of neuropeptide B/W signaling system have been predominantly detected and mapped within the CNS. In the rat, this system includes neuropeptide B (NPB), neuropeptide W (NPW) and their specific receptor NPBWR1. This signaling system has a wide spectrum of functions including a role in modulation of inflammatory pain and neuroendocrine functions. Expression of NPB, NPW and NPBWR1 in separate heart compartments, dorsal root ganglia (DRG) and stellate ganglia was proven by RT-qPCR, Western blot (WB) and immunofluorescence. Presence of mRNA for all tested genes was detected within all heart compartments and ganglia. The presence of proteins preproNPB, preproNPW and NPBWR1 was confirmed in all the chambers of heart by WB. Expression of preproNPW and preproNPB was proven in cardiac ganglionic cells obtained by laser capture microdissection. In immunofluorescence analysis, NPB immunoreactivity was detected in nerve fibers, some nerve cell bodies and smooth muscle within heart and both ganglia. NPW immunoreactivity was present in the nerve cell bodies and nerve fibers of heart ganglia. Weak nonhomogenous staining of cardiomyocytes was present within heart ventricles. NPBWR1 immunoreactivity was detected on cardiomyocytes and some nerve fibers. We confirmed the presence of NPB/W signaling system in heart, DRG and stellate ganglia by proteomic and genomic analyses.

## 1. Introduction

Neuropeptide B (NPB) and neuropeptide W (NPW) are structurally and functionally related endogenous neuropeptides. NPB peptide is produced by proteolytic cleavage from a preproNPB molecule, and two NPB isoforms, NPB23 and NPB29 (consisting of 23 and 29 amino acids, respectively) can be produced in humans. However, only NPB29 has been described in nonhuman species. Similarly, two isoforms of NPW have been observed, NPW23 and NPW30, which are produced from a common precursor, preproNPW. Abundance of both NPW isoforms has been demonstrated in several species, including human, rat, mouse, pig and chicken. Biological activities of NPB and NPW are mediated by orphan G-protein-coupled receptors, GPR7 (also called NPBWR1) in humans, rats and mice and GPR8 (NPBWR2) in humans only. NPB and NPW together with these receptors constitute NPB/W signaling system [1].

The existence of both peptides was proven relatively recently, namely in 2002 [2,3], while both receptors, GPR7 and GPR8, were described earlier [4]. Therefore, comprehensive information on the presence or absence of these peptides/proteins in individual organs or tissues is not yet available. Since NPB and NPW are neuropeptides, their expression and distribution have been studied primarily in the CNS. Some authors detailed the distribution of individual members of the NPB/W signaling system within the CNS at both gene expression and protein levels. Neurons containing NPB and/or NPW are localized in several regions of the brain, including the hypothalamus, hippocampus, limbic system, midbrain and brain stem [5,6,7,8,9]. Furthermore, information on gene expression of these proteins in homogenates of some tissues in pig and chicken, including heart, is also available [10,11]. In most cases, these data were obtained mainly by RT-PCR, so nothing indicates the tissue distribution of this signaling system in the periphery.

The aim of this study was to identify and confirm the presence of the members of NPB/W signaling system in separate compartments of rat heart by genomic and proteomic analyses. Additionally, thoracic dorsal root ganglia (DRG) and stellate ganglia were included in the evaluation in order to examine potential expression of the NPB/W signaling system in sympathetic and sensory nerve cell bodies innervating the heart. The identification of NPB, NPW and its receptor NPBWR1 may provide new insights and a platform for exploring the relationship of NPB/W signaling in the regulation of heart functions.

## 2. Results

### 2.1. Detection of mRNAs of the Members of NPB/W Signaling System by RT-qPCR

**Figure 1:**
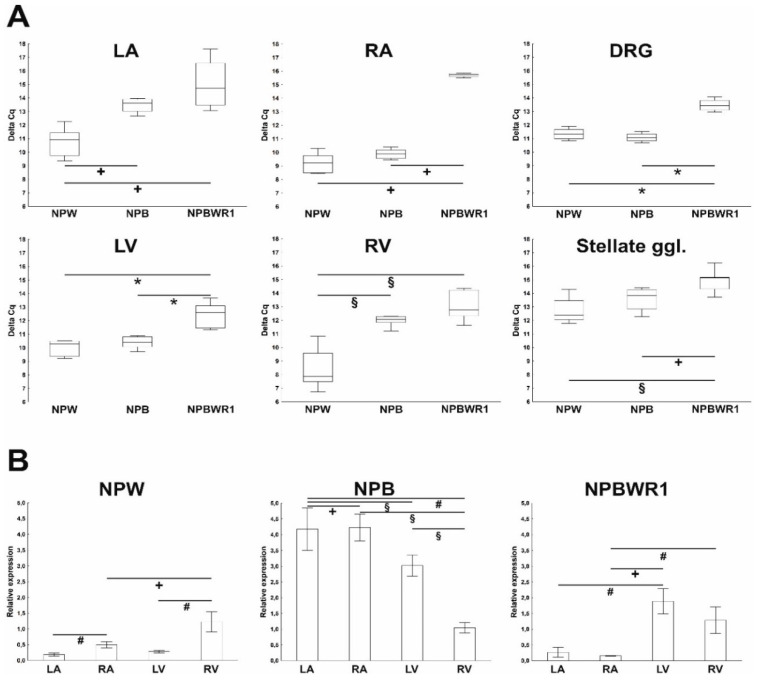
Quantitative RT-PCR analyses of neuropeptide B (NPB), neuropeptide W (NPW), and NPBWR1 mRNAs in heart chambers of rats. (**A**) Comparison of expression of studied genes within each heart compartment, dorsal root ganglia (DRG) and stellate ganglia. Data are presented as ΔCq values (compared to β-actin) to indicate differences in expression between different targets. Hence, low values represent high expression. Statistically significant differences are shown graphically (Mann–Whitney test; *n* = 5–8 in each group). (**B**) Comparison of separate heart compartments: left atrium (LA), right atrium (RA), left ventricle (LV) and right ventricle (RV). Data are presented as relative expression ± SEM. Mean values from RV were used as a calibrator and were settled as 1. Statistically significant differences between the heart compartments are shown graphically (Mann–Whitney test; *n* = 5–8 in each group). ^#^ *p* < 0.05, **^+^** *p* < 0.01, **^§^** *p* < 0.005, * *p* < 0.001.

Presence of mRNA for preproNPB, preproNPW and NPBWR1 was detected in all heart compartments, i.e., left atrium (LA), right atrium (RA), left ventricle (LV) and right ventricle (RV), as well as in DRG and stellate ganglia. However, very high Cq values oscillating in the range of 29–34 for all tested genes indicate their low level of expression. Statistical analysis of Cq values of studied genes within each separate heart compartment, DRG and stellate ganglia revealed that in the LA and RV, NPB and NPBWR1 mRNAs were expressed less than the gene for NPW. In RA and LV, NPBWR1 gene was expressed in a lesser extent than genes for NPB and NPW (Figure 1A). In the DRG and stellate ganglion, the level of mRNA expression of genes for NPB and NPW did not differ significantly, but both were expressed more than mRNA for NPBWR1 (Figure 1A). Furthermore, for better understanding, expression in LA, RA and LV was compared with expression in RV by assigning expression level of RV to 1 and setting expression in other heart chambers against it. Expression of mRNA for NPB was highest in RA and lowest in RV; mRNA expression for NPW was highest in RV and lowest in LA, mRNA expression for NPBWR1 was higher in the ventricles than in the atria (Figure 1B). Generally, the differences in the expression of studied genes between separated heart chambers were relatively small, not even reaching statistical significance in some cases. 

### 2.2. Identification of Peptides/Proteins of NPB/W Signaling System by WB

**Figure 2:**
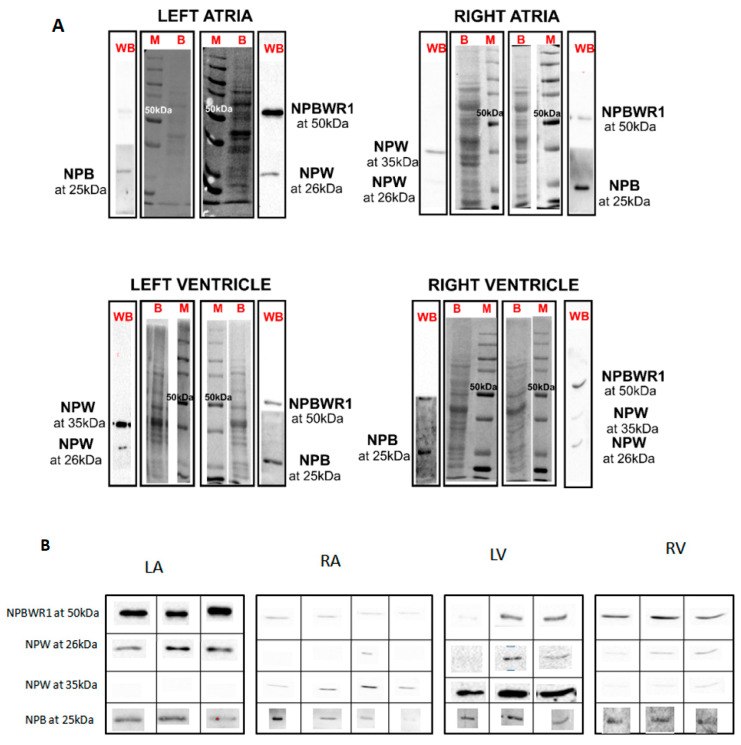
Identification of NPB, NPW and NPBWR1 by Western blot. Western blot (WB) analyses were done using commercially available antibodies against NPB (1:200), NPW (1:1000) and NPBWR1 (1:2000). Twenty-five micrograms of protein was used from each heart chamber: left atria (*n* = 3), right atria (*n* = 4), left ventricle (*n* = 3) and right ventricle (*n* = 3). We observed bands at 50 and 25 kDa for NPBWR1 and NPB, respectively. Two bands were observed for NPW at 35 and 26 kDa. (**A**) Lane WB represents immunoblot signals; lane B represents total protein transferred onto nitrocellulose membrane after Ponceau staining; lane M represents molecular weight marker. (**B**) Bands obtained from tested samples.

**Figure 3:**
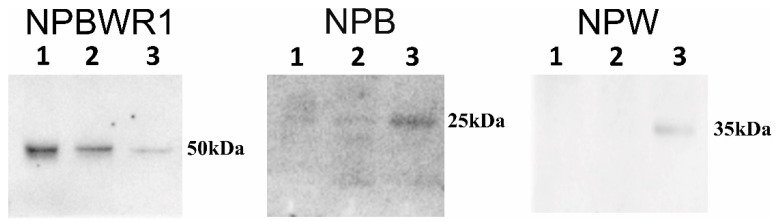
Identification of NPB, NPW and NPBWR1 by Western blot. WB analyses were done using commercially available antibodies against NPB (1:200), NPW (1:1000) and NPBWR1 (1:2000). Twenty-five micrograms of protein was used from dorsal root ganglion (DRG) and stellate ganglia. Lane 1: DRG (pool of *n* = 5); Lane 2: stellate ganglion (pool of *n* = 5); Lane 3: spinal cord.

Good protein profiling of rat heart lysate was visible on SDS-PAGE. Similar amounts of proteins were transferred to nitrocellulose membrane and used for detection of NPB/W signaling system through Western blot (WB) analysis. Single immunogenic bands were detected for NPBWR1 and NPB at 50 and 25 kDa, respectively. Two immunogenic bands were observed for NPW at 35 and 26 kDa (Figure 2). Immunogenic bands of NPBWR1, preproNPB and preproNPW were identified from different heart chambers such as LA (*n* = 3), RA (*n* = 4), LV (*n* = 3) and RV (*n* = 3) and ganglia such as DRG (pool of five) and stellate ganglia (pool of five). Immunogenic bands of NPBWR1 and preproNPB were present in all the chambers of rat heart (Table 1. An immunogenic band of preproNPW at 35 kDa was present in all the chambers of rat heart except LA. On the contrary, the presence of an immunogenic band of preproNPW at 26 kDa was observed in one out of four and one out of three rats in RA and LV, respectively. Additionally, a strong immunogenic band of NPBWR1 was detected for DRG and stellate ganglia (Figure 3); however, a very weak band for preproNPB and no band for preproNPW was observed in the same tissues (Table 1).

### 2.3. Validation of Anti-NPB, Anti-NPW and Anti-NPBWR1 Antibodies

#### 2.3.1. Pre-adsorption Assay using the Synthetic Peptides

To validate the commercial antibodies, we performed a pre-absorbance assay. Highly immunogenic peptide sequences were selected and synthesized for pre-absorption assay. We used identical peptide sequences (species-specific) that were used as an immunogen to raise the respective antibodies. Moreover, we also observed 93% sequence homology in selected NPB peptide mouse versus rat, 97% sequence homology in selected NPW peptide human versus rat and 79% sequence homology in selected NPBWR1 human versus NPBWR1 rat (Table 2). All the antibodies used in the experiment were cross-reactive to rat species. Therefore, we synthesized the peptide sequence of NPW and NPBWR1 from human and NPB from mouse, not from rat.

**Figure 4:**
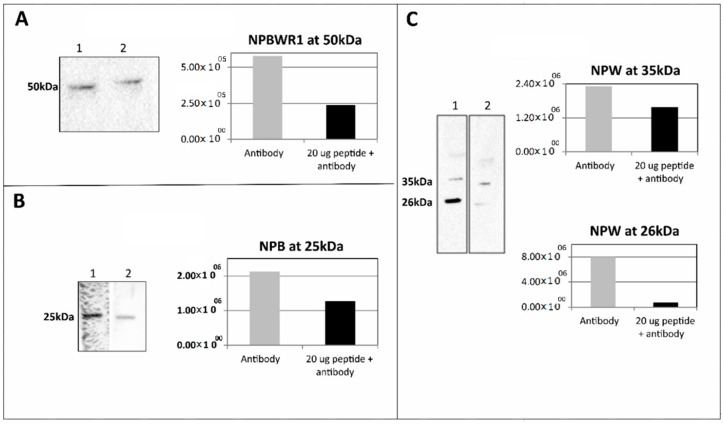
Pre-adsorption assay: (**A**) Here, 12.5 µg of rat heart lysate (RV) was used. Lane 1 was incubated with anti- NPBWR1 (1:2000) and Lane 2 was incubated with pre-adsorbed anti- NPBWR1 (1:2000) with 20 µg of peptide NPBWR1 (33–62). (**B**) Here, 50 µg of rat heart lysate (RV) was used. Lane 1 was incubated with anti-NPB (1:400) and Lane 2 was incubated with pre-adsorbed anti-NPB (1:400) with 20 µg of peptide NPB (22–50). (**C**) Here, 25 µg of rat heart lysate (RV) was used. Lane 1 was incubated with anti-NPW (1:1000) and Lane 2 was incubated with pre-adsorbed anti-NPW (1:1000) with 20 µg of peptide NPW (32–66).

Immunoreactive proteins identified on WB were further examined by using the pre-adsorption assay. Overnight pre-adsorption of the primary antibody with the corresponding synthetic peptide prevented the recognition of NPBWR1 protein in WB, but not completely (Figure 4A). Due to the lower reactivity/signal ratio of anti-NPB towards heart lysate and high background issued with 1:200, 50 µg of protein was used with anti-NPB (1:400). Overnight pre-absorption of the primary antibody with the corresponding synthetic peptide prevented the recognition of preproNPB protein in WB (Figure 4B).

Overnight pre-adsorption of the primary antibody with the corresponding synthetic peptide prevented the recognition of preproNPW protein at 35 and 26 kDa. The band observed at 26 kDa showed the best depletion among all the peptides used. However, the band at 35 kDa was only partially depleted (Figure 4C). The percentage of inhibition was calculated by using the following Formula (1):

(1) $$\%\;\mathrm{Inhibition}\;=\;\frac{\mathrm{WB}\;\mathrm{signal}\;\mathrm{without}\;\mathrm{peptide}-\mathrm{WB}\;\mathrm{signal}\;\mathrm{with}\;\mathrm{peptide}}{\mathrm{WB}\;\mathrm{signal}\;\mathrm{without}\;\mathrm{peptide}}\;\times \;100$$

More than 90% inhibition of anti-NPW at 26 kDa, 31.6% inhibition of anti-NPW at 35 kDa, 40.1% inhibition of anti-NPB at 25 kDa and 58.7% inhibition of anti-NPBWR1 at 50 kDa were observed (Supplementary Figure S1).

#### 2.3.2. Immunofluorescence Analysis of NPB, NPW and NPBWR1 Localization

**Figure 5:**
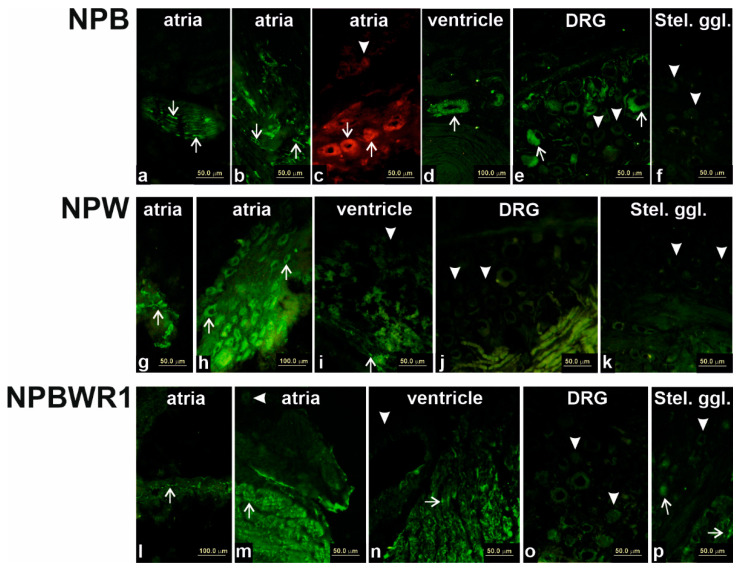
Immunofluorescence for neuropeptide B (NPB; **a**–**f**), neuropeptide W (NPW; **g**–**k**) and their receptor 1 (NPBWR1; **l**–**p**) in the rat heart compartments, dorsal root ganglia (DRG) and stellate ganglia. Antiserum against NPB exerts specific immunoreactivity (IR) in nerve fibers (some of them marked by arrows), cardiac ganglionic cells within heart atria (arrows) and smooth muscle cells of coronary vessels (arrow). Some neurons within DRG exert specific NPB immunoreactivity (IR; arrow), while some of them do not (arrowheads). Neurons within stellate ganglion do not show any specific reaction with NPB antiserum (arrowheads). Within the heart, NPW-IR is visible in some nerve fibers and bodies of intracardiac neurons (arrows). Neurons of DRG and stellate ganglion do not exert specific IR with NPW antiserum (arrowheads). NPBWR1 antiserum shows IR in cardiomyocyte cell membranes (some marked by arrows), some (arrow) but not all (arrowheads) neurons within stellate ganglia and some nerve fibers within stellate ganglia (arrows). No specific IR is present within the DRG. *n* = 3 of each tissue.

Specific NPB immunoreactivity (IR) was detected within the heart, mainly in nerve fibers. These NPB immunoreactive nerve fibers were more numerous within the atria, where they were localized in between atrial cardiomyocytes, in the vicinity of nerve cell bodies within heart ganglia and within nerve fiber bundles (Figure 5a–c). Additionally, some but not all nerve cell bodies within heart ganglia exerted specific NPB-IR (Figure 5c). Smooth muscle cells of some coronary arteries exerted weak reactivity with NPB antibody, while cardiomyocytes showed no specific NPB-IR (Figure 5d). Some but not all nerve cells bodies and nerve fibers showed NPB-IR within DRG (Figure 5e). In the stellate ganglia, only few nerve fibers within nerve bundles exerted NPB-IR and few nerve cell bodies showed weak IR (Figure 5f).

Within heart atria, NPW-IR was visible in the nerve cell bodies as well as in the nerve fibers of heart ganglia (Figure 5g,h). Very weak nonhomogenous staining of cardiomyocytes was present within heart ventricles (Figure 5i). No specific reaction with NPW antisera was detected in DRG (Figure 5j) and stellate ganglia (Figure 5k).

Specific NPBWR1-IR was visible on the surface of atrial as well as ventricular cardiomyocytes (Figure 5m,n), although the intensity of immunofluorescence was not homogenous throughout the heart. Additionally, some nerve fibers within heart atria showed specific reaction with NPBWR1 antisera (Figure 5i). No specific labeling was observed in smooth muscle cells of coronary circulation (Figure 5n). No specific reaction with NPBWR1 antisera was detected in DRG (Figure 5o), and only NPBWR1-IR nerve fibers were detected in stellate ganglia (Figure 5p).

### 2.4. LCM Analysis of NPB and NPW mRNA Expression within the Heart

Expression of mRNAs for NPB and NPW was detected in some individual cell types of heart tissue, nerve cell bodies of intracardiac nervous system (GCs), cardiomyocytes (CMs) and smooth muscle cells (SMCs). These cells were isolated by means of laser capture microdissection (LCM) and acquired samples were analyzed by RT-qPCR. In total, 19 GC, 4 CM and 7 SMC samples were prepared and analyzed. Expression of NPB mRNA was detected in 8 GC, 1 SMC and 0 CM samples. NPW mRNA expression was detected only in 1 GC sample and in no samples of other tested cell types.

## 3. Discussion

According to our knowledge, this is the first study demonstrating the presence and tissue distribution of members of NPB/W signaling system within separate compartments of rat heart and ganglia innervating it at both genomic and proteomic levels.

The presence and tissue distribution of individual members of the NPB/W signaling system were studied by several working groups in the central nervous system, and detailed information is available in the literature [1]. It has been suggested that the NPB/W signaling system is involved in several central regulations including regulation of neuroendocrine functions; regulation of feeding activity; autonomic regulation; and activation of stress axis, pain sensation and energy homeostasis. Additionally, it plays a role in emotions, anxiety and fear [12]. Despite the action throughout common receptor, NPB and NPW exert some differences in their biological activities [1].

Relatively little is known about the localization of this signaling system within the peripheral nervous system and/or other tissues. Expression of members of this signaling system at mRNA level has been more or less comprehensively studied in human, rat, pig and chicken peripheral tissues, confirming the presence of mRNA for individual members of the NPB/W signaling system, for example in kidneys and organs of respiratory system, urogenital system and gastrointestinal tract. The presence of mRNA for NPB was also demonstrated in whole-heart RNA in human, chicken and rat; for NPW in chicken and pig; and for NPBWR1 in pig only [2,10,11,13]. In this study, the heart was divided into individual chambers in order to obtain more precise information about expression and distribution of members of NPB/W signaling system, as cardiac tissue is not homogenous in terms of the content of different cell types in the individual cardiac compartments, especially with respect to the localization of the intracardiac nervous system. The results of the qPCR analysis show an overall very low level of expression of individual genes of the investigated signaling system. The level of NPB mRNA expression was higher in atria than in ventricles, suggesting predominant neuronal localization, as nerve cell bodies of the intracardiac nervous system are localized only within the atria in rats [14]. This assumption was confirmed by immunofluorescence analysis, which showed specific NPB and NPW-IR in the bodies of intracardiac ganglion neurons. This finding indicates the functioning of these peptides in the heart as neurotransmitters. However, compared to the expression of the most abundant neuropeptide transmitter of intracardiac neurons, NPY, the expression of these neuropeptides is about a thousand times lower [15]. By means of WB analysis, the presence of NPB and NPW peptides was proven in each heart chamber. Results of qPCR showed expression of NPB as well as NPW genes in the heart ventricles, which also indicates non-neuronal expression of these genes. However, the total amounts of NPB mRNA and protein detected in ventricles were low, just at the limit of detectability. The differences in NPB and NPW gene expression between the right and left ventricles cannot be explained by the different proportions of myocytes and fibroblasts, as the percentages of these cell types were found to be similar in both ventricles [16]. However, differences in the proportion of endothelial cells or smooth muscle cells could be considered, given that the results of our immunofluorescence analysis show the presence of NPB in smooth muscle cells and suggest that smooth muscle cells are probably the main non-neuronal source of NPB in the heart.

The expression of NPW mRNA and peptide was also demonstrated in the heart ventricles, but the source is not entirely clear. Immunofluorescence results indicate weak expression in cardiomyocytes, but due to the overall very low expression of this gene in the ventricles, its presence cannot be ruled out in endothelial cells too, where such a low amount would not be detectable by immunofluorescence. The case of expression and detection of NPY in the heart is similar. The expression of NPY in the rat heart was also demonstrated in non-neuronal cells, specifically in cardiomyocytes and endothelial cells [17,18]. However, the presence of preproNPY in endothelial cells cannot be reliably detected by immunofluorescence [15,19]. This is probably due to the NPY content in these cells being lower than the immunofluorescence detection limit.

RT-qPCR analysis of RNA from samples obtained by LCM was done in order to prove the expression of NPB and NPW in individual cell types. We have shown that some intracardiac neurons express mRNA for NPB and/or NPW. We also demonstrated the expression of NPB mRNA in vascular smooth muscle cells. Unfortunately, we were unable to demonstrate the expression of these genes in cardiomyocyte cells. This could be due either to the absence of expression of these genes in cardiomyocytes or, possibly, to their very low expression level, which cannot be detected in such a small sample. A limitation in the determination of the expression of the monitored genes in the sample of cardiomyocytes obtained by LCM is the impossibility of excluding the presence of endothelial cells and fibroblasts. In fact, in a tissue section stained with hematoxylin, it is not possible to distinguish between these cells and cardiomyocytes and remove them from the sample. Absence of WB signal in some protein samples could be also caused by low amount of studied proteins.

Another source of NPB in the heart could be nerve fibers coming from the DRG, as the majority of DRG neurons expressing NPB are visible from immunofluorescence experiments. On the contrary, neurons within the stellate ganglion contain very small amounts of NPB, which leads to assumption that sympathetic fibers innervating the heart are not the source of NPB. NPW probably does not enter the heart via sympathetic or sensory nerves because we have failed to demonstrate the presence of this peptide in the ganglia innervating the heart.

The regulation of NPBW signaling system is still only partially disclosed. Within the central nervous system, NPW neurons could be directly or indirectly stimulated by osmotic stimuli [20], stress [21] and/or nerve growth factor and brain derived neurotrophic factor [22]. Additionally, NPBWR1 mRNA expression is regulated by leptin, NPW and ghrelin in the neurons of nodose ganglia [23]. NPB is involved in sleep regulation [1]. 

So far, we can only speculate about the function of this signaling system in the heart, because very little information is currently available in the literature. It has been demonstrated that NPB/W signaling system is involved in the regulation of blood pressure both at the central and peripheral levels [1]. Our finding demonstrating the presence of NPB within smooth muscle cells of coronary circulation supports the idea that NPB could be involved in regulation of coronary circulation. In addition, both neuropeptides described in this study are present in the bodies of some intracardiac neurons, suggesting their possible involvement in the regulation of cardiac function. However, this hypothesis must be confirmed by a functional study.

Finally, this report confirms the presence of neuropeptide W/B signaling system in all the chambers of rat heart as well as in DRG and stellate ganglia. However, the function of this signaling system in the heart is a matter for further investigation.

## 4. Materials and methods

### 4.1. Experimental Animals

Adult male lean Zucker rats (body weight about 230 g) were used (*n* = 16). The animals were housed 2 per cage and fed ad libitum with free access to drinking water. Body weights were measured once a week. All experiments were approved by the University Committee for Experiments on Laboratory Animals and Ministry of Education, Youth and Sports of the Czech Republic (MSMS-10669/2016-6; 15. 3. 2016) and were conducted in accordance with the “Guide for the Care and Use of Laboratory Animals” (NIH Publication No. 85-23, revised 1996) as well as the relevant Guidelines of the Czech Ministry of Agriculture for scientific experimentation on animals. 

Rats were sacrificed by decapitation. Hearts were rapidly excised; rinsed with ice-cold saline solution; freed of connective tissue and fat and divided into the left atrium with the interatrial septum (LA), right atrium (RA), and free walls of left (LV) and right (RV) ventricles; embedded in optimum cutting temperature compound (Takara, Mountain View, CA, USA); and frozen in precooled isopentane (for immunofluorescence and/or for laser capture microdissection) or directly frozen in liquid nitrogen (for RNA and/or protein isolation). A similar procedure was used to prepare DRG, stellate ganglia and thoracic spinal cord (SC) samples. Samples were kept at −80 °C until use. Eight animals were used for RNA isolation and subsequent RT-qPCR, 5 animals for protein isolation with subsequent Western blotting and 3 animals for immunofluorescence and LCM.

### 4.2. Extraction of Protein

Tissues (*n* = 3–5 of each) were weighed and minced into small pieces. PBS was added in a total amount of 1 µL per 1 μg of tissue. Samples were homogenized for 3 min with rest for 1 min in ice per cycle. In total, three cycles were used. Homogenate was centrifuged at 10,000× *g* for 15 min and debris was removed. Supernatant was collected and protein content was estimated by Bradford dye. For WB analysis, 25 μg of protein was used. 

### 4.3. SDS-PAGE and WB Analysis 

Isolated proteins were subjected to SDS-PAGE 10% under reducing condition (2% *v*/*v* β-mercaptoethanol). Gel was transferred onto nitrocellulose membrane (0.2 μm; Bio-Rad, Hercules, CA, USA) at 16 V overnight at 4 °C. The membrane was blocked with 5% milk powder in PBST (PBS pH 7.4, 0.1% TWEEN 20) for 1 h and incubated with anti-NPB (1:200; Thermo Fischer Scientific, Waltham, MA, USA), anti-NPW (1:1000; Bioss Antibodies Inc, Boston, MA, USA) and anti-NPBWR1 (1:2000; Bioss Antibodies Inc, USA) for 1 h at room temperature. The membrane was washed three times (10 min per wash) with PBST and incubated with HRP-conjugated anti-IgGs (1:4000) for 45 min at room temperature. The membrane was washed three times as mentioned above with PBST and blots were developed using an ECL kit (Advansta, San Jose, CA, USA). Data were analyzed in Image Lab software (Bio-Rad). Samples isolated from the spinal cord served as a positive control.

### 4.4. Selection of Neuropeptides NPB, NPW and NPBWR1

UniProtKB/Swiss-Prot protein database was used to select the peptide sequences. Accession numbers for NPB (Mouse), NPBWR1 (Human) and NPW (Human) were Q8K4P1, P48145 and Q8N729, respectively. Peptide sequences of mouse NPB (22-50), human NPBWR1 (220–250) and human NPW (33–62) were selected (Table 3). Basic Local Alignment Search Tool (BLAST) was used for comparing primary biological sequence information in order to find alignment of the sequences of different species. Moreover, availability of antibodies was also confirmed, where selected peptides were used as immunogens for respective antibody production with cross-reactivity with rat species. 

### 4.5. Selection of Neuropeptide Sequences for Peptide Synthesis 

A bioinformatics approach was used to identify the highly immunogenic sequence of NPB, NPW and NPBWR1. BLAST (Basic Local Alignment Search Tool) was used to find alignment of the sequence of different species in the protein database. Three sequences were selected: mouse NPB (22–50), human NPBWR1 (220–250) and human preproNPW (33–62). The selection of peptide sequences to be synthesized was done according to the availability of the corresponding antibodies on the market, while selected sequences should be used to produce the antibody used for experiments. 

### 4.6. Peptide Synthesis and Purification

Highly immunogenic peptides, i.e., NPB (22–50), NPW (220–250) and NPBWR1 (33–62) were synthesized in solid phase using the automated microwave-assisted peptide synthesizer Liberty Blue (CEM Corporation, Matthews, NC, USA) following the standard protocols for Fmoc/tBu strategy. The peptides were purified by semipreparative RP-HPLC on a Waters instrument (Separation Module 2695, detector diode array 2996) using a Phenomenex (Torrance, CA, USA) Jupiter column C18 (10 μm, 250 × 10 mm) at 4 mL/min with solvent systems A (0.1% TFA in H_2_O) and B (0.1% TFA in CH_3_CN). The purity of the peptides was analyzed by analytical HPLC using a Waters ACQUITY HPLC coupled to a single-quadrupole ESI-MS (Waters 3100 Mass Detector) supplied with a BEH C18 (1.7 μm 2.1 × 50 mm) column at 35 °C at 0.6 mL/min with solvent systems A (0.1% TFA in H_2_O) and B (0.1% TFA in CH_3_CN).

Data were acquired and processed using MassLynx software (Waters, Milford, MA, USA). The analytical data are reported in detail in Table 4.

### 4.7. Pre-Adsorption Assay Using the Synthetic Peptides

Primary antibodies anti-NPW (1:1000), anti-NPB (1:400) and anti-NPBWR1 (1:2000) were incubated separately with 20 µg of the synthetic peptides preproNPW (33–62), preproNPB (22–50) and NPBWR1 (220–250), respectively at 4 °C overnight. Tissue lysate from RV (25 µg of protein for anti-NPW, 50 µg of protein for anti-NPB and 12.5 µg of protein for anti-NPBWR1) was used for pre-adsorption WB analysis. The methodology used for WB analysis was similar to that mentioned above. 

### 4.8. Laser Capture Microdissection (LCM)

The whole procedure was done according to a protocol published earlier [24]. Briefly, sections of heart atria were stained with alum hematoxylin solution, washed with water and dehydrated with ethanol. Intracardiac neurons, vascular smooth muscle cells and cardiomyocytes were collected from the sections. Subsequently, RNA was isolated from obtained samples using RNeasy Micro Kits (Qiagen, Hilden, Germany) according to the manufacturer’s instructions. 

### 4.9. RNA Isolation and RT-qPCR Analysis

Total RNA was isolated from all four heart compartments (*n* = 8 of each) using TRI reagent (Sigma, St. Louis, MO, USA) following the protocol of the manufacturer. Contaminating DNA was destroyed with 1 U DNAse/μg of total RNA (Invitrogen, Carlsbad, CA, USA). RNA was reverse-transcribed using Superscript III Reverse Transcriptase (Invitrogen) for 50 min at 42 °C. Single-strand cDNA was synthesized from 3 μg of total RNA. The qPCR analysis was done as described previously [12]. The primers were designed to amplify the sequence corresponding to nucleotides 207–310 (forward: GATGCGCCCAAGCGTAAGAA; reverse: TACACTGGAAAGTCCCTCGG) of the published rat preproNPB cDNA sequence (Genbank Accession No. NM_153293.1), nucleotides 538–637 (forward: GCTAGAGCCTTCGGTGAGAC; reverse: ATCGGTTCTTGAGACGGTCG) of the published rat preproNPW cDNA sequence (Genbank Accession No. NM_153294), nucleotides 625–712 (forward: ACTCTAGTGTTGGGCTTCGC; reverse: CTAGCTGGATAGCACGCAGT) of the published rat NPBWR1 cDNA sequence (Genbank Accession No. NM_001014784.1) and nucleotides 821–1029 (forward: TTCCTTCCTGGGTATGGAATC; reverse: GTTGGCATAGAGGTCTTTACGG) of the published rat β-actin cDNA sequence (Genbank Accession No. NM_031144).

Real-time PCR was performed in the iCycler (Bio-Rad, Prague, Czech Republic). Final assay volumes were 15 μL and contained 7.5 μL iQ SYBR Green Supermix (Bio-Rad, Prague, Czech Republic), 0.15 μL of each primer (20 nmol/L), 3 μL of diluted cDNA and 4.2 μL of ultrapure water. The quantitative PCR reactions were performed as follows: denaturation at 95 °C for 10 min followed by 45 cycles of amplification (95 °C for 20 s, 60 °C for 20 s and 72 °C for 20 s). Each run was completed with a melting curve analysis in order to confirm the specificity of amplification and lack of primer dimers. Each pair of primers yielded a single peak in the melting curve and a single band of the expected size in agarose gel. Reactions for all samples were performed in triplicate. Standard curves were generated for each pair of primers using 10-fold serial dilution of standards. Blank controls with the omitted template were used. NPB, NPW and NPBWR1 mRNAs were determined in all heart chambers by subtracting their quantitative cycle values (Cq) to Cq of reference gene. As it exerts good stability within the heart, β-actin was used as a reference gene. The relative expression ratios were calculated using the 2^−ΔΔCq^ method.

### 4.10. Immunofluorescence

Shock-frozen hearts, stellate ganglia and/or DRG (*n* = 3 of each) were cut into 10-μm-thick sections using a Leica CM1850 cryostat (Leica, Bensheim, Germany) and fixed for 10 min in cold acetone. Samples were exposed at room temperature for 1 h to the medium blocking the nonspecific binding sites (1% BSA, 0.1% Triton X-100 and 2% normal goat serum) and then incubated with anti-NPB (1:200; Thermo Fischer Scientific, Waltham, MA, USA), anti-NPW (1:1000; Bioss Antibodies Inc, Woburn, MA, USA) and anti-NPBWR1 (1:2000; Bioss Antibodies Inc, USA) overnight at 4 °C. The samples were washed three times (10 min per wash) with PBS and incubated with FITC-conjugated anti-IgGs (1:400). The incubation lasted for 2 h at room temperature in the dark. After drying, coverslips were added to samples with one drop of DABCO (Sigma, St. Louis, MO, USA) mounting medium. Samples were observed with an Olympus BX 60 fluorescent microscope (Olympus, Prague, Czech Republic) equipped with appropriate filter combinations.

### 4.11. Statistical Analysis

All data are expressed as the mean ± standard error of the mean (SEM). All the data obtained from the individual experimental groups were first subjected to Shapiro–Wilk normality test. As the results of this test showed that the values in the individual groups did not show a normal distribution, a nonparametric Mann–Whitney test was subsequently used for statistical evaluation. Values of *p* < 0.05 were considered statistically significant. The analysis was performed using the software package STATISTICA Cz, version 7 (StatSoft CR, Prague, Czech Republic).

## Figures and Tables

**Table 1:** NPB/W signaling in rat heart.

NPB/W Signaling	LA (*n* = 3)	RA (*n* = 4)	LV (*n* = 3)	RV (*n* = 3)	DRG (Pool of *n* = 5)	SG (Pool of *n* = 5)
NPB at 25kDa	3/3	3/4	3/3	3/3	Faint band	Faint band
NPW at 26kDa	3/3	1/4	2/3	3/3	No band	No band
NPW at 35kDa	0/3	4/4	3/3	3/3	No band	No band
NPBWR1 at 50kDa	3/3	4/4	2/3	3/3	Strong band	Strong band

**Table 2:** Comparison of sequence homology of synthetic peptides NPW (33–62) human, NPB (22–50) mouse and NPBWR1 (220–250) human with NPW (33–62), NPB (22–50) and NPBWR1 (220–250) in rat.

Amino Acid Sequence	Peptide	Identifies	Homology
WYKPAAGPHHYSVGRASGLLSSFHRFPST	Mouse NPB (22–50)	27/29	93%
WYKPAAGSHHYSVGRAAGLLSSFHRFPST	Rat NPB (22–50)		

WYKHVASPRYHTVGRAAGLLMGLRRSPYLW	Human NPW (33–62)	30/31	97%
WYKHVASPRYHTVGRASGLLMGLRRSPYLW	Rat NPW (33–62)		
			
CVLYTTLLCRLHAMRLDSHAKALERAKKRVT	Human NPBWR1 (220–250)	23/29	79%
CALYI TLLCRLRA I QLDSHAKALDRAKKRVT	Rat NPBWR1 (220–250)		

**Table 3:** Immunogenic neuropeptide sequences of NPB, NPBWR1 (GPR7) and NPW.

Peptide	Sequence
Mouse NPB (22–50)	WYKPAAGPHHYSVGRASGLLSSFHRFPST
Human NPBWR1 (220–250)	CVLYTTLLCRLHAMRLDSHAKALERAKKRVT
Human NPW (33–62)	WYKHVASPRYHTVGRAAGLLMGLRRSPYLW

**Table 4:** Analytical data of the synthetic immunogenic neuropeptides.

S. No.	Neuropeptide	*R*t (min)	ESI-MS *m/z* Observed [M+3H]^3+^ (Calculated [M+3H]^3+^)	HPLC Purity
1	mouse NPB (22–50)	1.65	1071.82 (1072.5)	>98%
2	human NPBWR1 (220–250)	2.08	1199.71 (1200)	>98%
3	human NPW (33–62)	1.82	1181.18 (1182)	>95%

Analytical HPLC gradients at 0.6 mL min^−1^; solvent system A: 0.1% TFA in H_2_O; solvent system B: 0.1% TFA in CH_3_CN; A: 0‒90% B in 10 min.

## Supplementary Materials

The following are available online at https://www.mdpi.com/1422-0067/21/21/7827/s1, Figure S1: Pre-adsorption Assay: Inhibition of commercially purchased antibodies by the synthetic peptides NPB (1-29), GPR7 (220-250), and NPW (33-62).
