# Supplementary material for: Identification of NPB, NPW and Their Receptor in the Rat Heart
Source: Int J Mol Sci. 2020 Oct 22;21(21):7827. doi: 10.3390/ijms21217827 (PMC7659951; doi:10.3390/ijms21217827)
Supplement: Supplementary file 1 [file ijms-21-07827-s001.pdf]

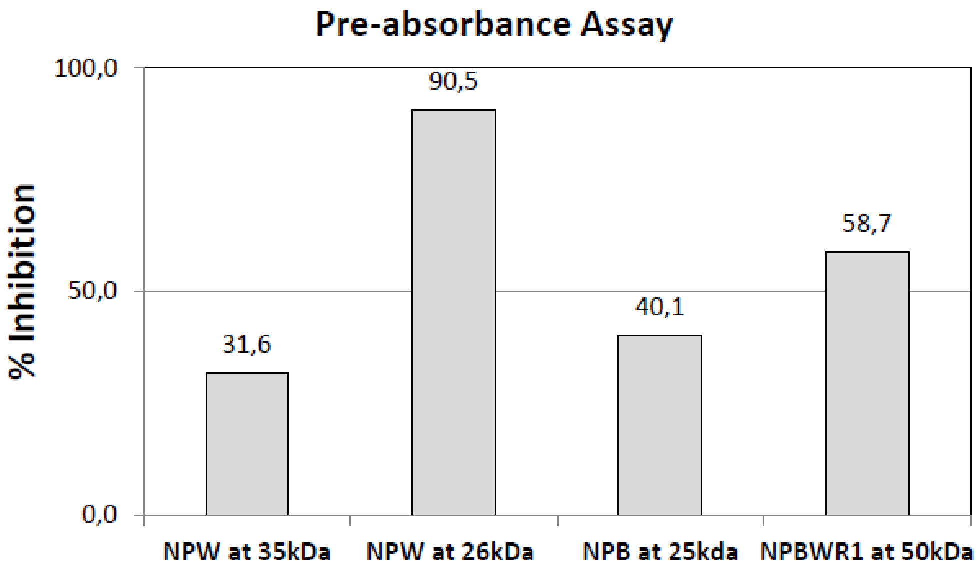

Supplementary FigureS1: Pre-adsorption Assay: Inhibition of commercially purchased antibodies by the synthetic peptides NPB (22–50), GPR7 (220–250), and NPW (33–62).
